# *Aloe vera*: A Sustainable Green Alternative to Exclude Antibiotics in Modern Poultry Production

**DOI:** 10.3390/antibiotics12010044

**Published:** 2022-12-27

**Authors:** Rifat Ullah Khan, Shabana Naz, Davide De Marzo, Michela M. Dimuccio, Giancarlo Bozzo, Vincenzo Tufarelli, Caterina Losacco, Marco Ragni

**Affiliations:** 1 Faculty of Animal Husbandry and Veterinary Sciences, College of Veterinary Sciences, The University of Agriculture, Peshawar 25000, Pakistan; 2 Department of Zoology, Government College University, Faisalabad 38000, Pakistan; 3 Department of Precision and Regenerative Medicine and Jonian Area, Section of Veterinary Science and Animal Production, University of Bari Aldo Moro, 70010 Bari, Italy; 4 Department of Veterinary Medicine, University of Bari Aldo Moro, 70010 Bari, Italy; 5 Department of Soil, Plant and Food Science, University of Bari Aldo Moro, 70126 Bari, Italy

**Keywords:** *Aloe vera*, alternative to antibiotics, poultry, production

## Abstract

Over the past 50 years, there has been a rapid increase in the need for poultry meat on a global scale to meet the rising demand from health, ecology, safety and equity. However, there has been a significant rise in recent years in both public demand and scientific interest for organic poultry farming, particularly when using medicinal herbs due to the rising concern of antibiotic resistance in end users. Ban on the use of antibiotics in the poultry industry has resulted in the demand of herbs as alternatives to antibiotics. Various research efforts have illustrated the nutritional value of *Aloe vera* in improving growth performance and immune status and acting as an antibacterial and anticoccidial agent in poultry. *Aloe vera* has been used as a supplement in the form of gel, alcoholic extract, powder, polysaccharide and aqueous extract. *Aloe vera* contains more than 200 nutrients, bioactive compounds, polysaccharides and saponins. In the current review, we have detailed the effect of *Aloe vera* as an alternative to antibiotics on growth performance, antimicrobial and antiparasitic activities and blood biochemical alternations in poultry.

## 1. Introduction

Poultry farming provides an affordable source of nutrients and has made significant contributions to the world’s food security. Despite recent advancements in efficiency, there is still a biosecurity gap because of infectious diseases and rising feed prices [1]. Additionally, due to the spread of infectious diseases, birds raised in intensive and super intensive systems are more vulnerable to diseases, resulting in large mortality and financial losses [2]. The adoption of well accepted integrated strategies including the administration of antibiotics, vaccines, and other treatments is therefore required [3]. However, chemotherapy is no longer allowed in many countries because of its negative effects on the environment and human health, including as the emergence of bacteria resistant to antibiotics, a decline in bird immunity, and security issues regarding people’s health. Due to the emergence of microbial resistance and the presence of microbial residues in meat and eggs, the use of antibiotics as growth promoters has recently been prohibited [1]. Since prohibiting the use of antibiotics in the production of chicken [2,3], there are strong arguments to keep using different methods. Antibiotic alternatives that are safer are thus highly encouraged for the control of infectious bacteria. Herbal therapy has historically been a useful therapeutic option in many parts of the world. However, little of this crucial ethno-veterinary information has been documented. In view of the rising cost of pharmaceuticals and their expanding significance in the future production of organic goods, herbal plants need to be recorded. Additionally, because herbs are naturally a broad spectrum, they offer a viable alternative when diseases develop resistance to antibiotics. Several feed additives have been proposed such as probiotic [3,4,5,6,7,8,9,10], prebiotics [11,12,13,14], enzymes [9,15,16,17,18], and botanicals [19,20,21,22,23,24,25,26,27,28,29,30,31,32,33,34,35,36,37,38,39] in poultry.

*Aloe vera* is a stemless, succulent medicinal plant belonging to the *Liliaceae* family with turgid, lance-shaped leaves with sharp points and jagged edges [40]. The Arabic word “Alloeh,” which means brilliant and bitter, is the source of the name *Aloe vera*. Physiologically, the most active member of the Aloe genus is *Aloe barbadensis* [41]. With more than 75 bioactive compounds, it is widely used in China, India, and Egypt. *Aloe vera* has been utilised for medicinal purposes since ancient times [42,43], and it is still used today to make cosmetics, medications, and foods for humans [44]. *Aloe vera* has been documented to be used by farmers in rural areas to control and treat poultry diseases [45].

**Figure 1:**
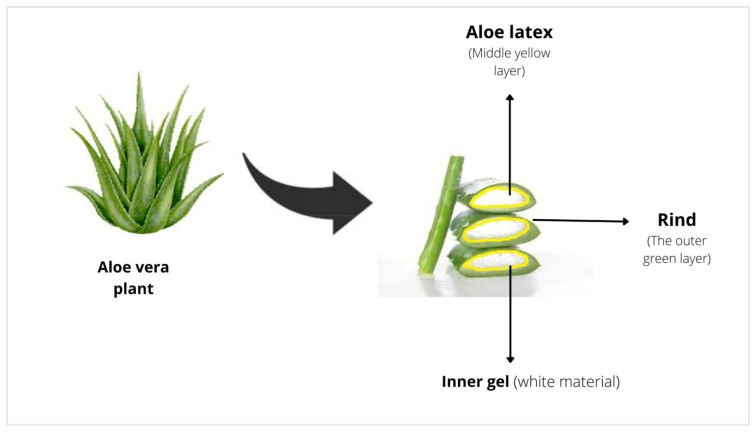
Gross morphology of the *Aloe vera* plant.

An *Aloe vera* plant may reach a mature height of 80 to 100 cm. The mature leaves are formed by three layers (Figure 1). The top layer, referred to as the rind, contains the phloem, xylem, and vascular bundles that transport nutrients. The intermediate layer known as “sap” (aloe latex) contains a bitter liquid that takes the form of a yellow substance. *Aloe vera* is primarily grown for the inner layer, which is a whitish, viscous, semisolid, transparent gel-like substance having therapeutic potentials [46].

**Figure 2:**
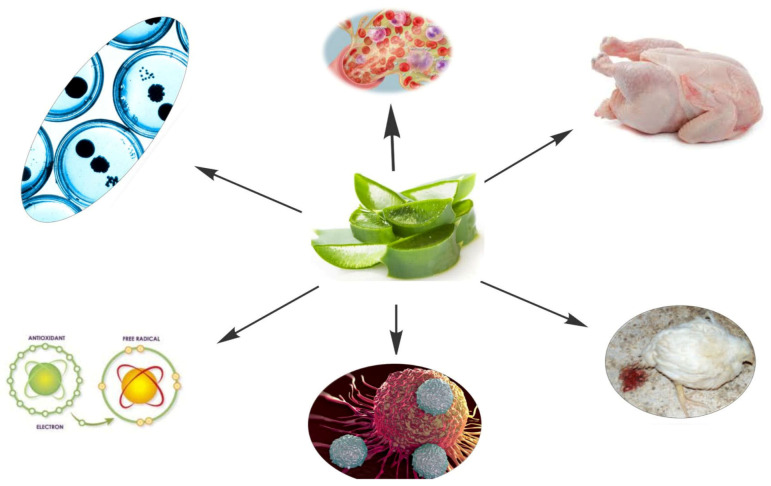
Multiple beneficial effects of *Aloe vera* in poultry.

Polysaccharides, phenolic substances, vitamins, minerals, sugars, proteins, and saponins make up its gel and have unique pharmacological properties in various disorders [47,48]. About 98% of the *Aloe vera* gel’s ingredients are water [41]. *Aloe vera* gel’s solid component has an average pH of 4.55 and is made up of 0.66% non-soluble material and 0.56% soluble material. There are more than 75 distinct active components in this substantial amount. Vitamin A, riboflavin, thiamine, pyridoxine, niacin, vitamin E, choline, vitamin C, and folic acid are among the vitamins abundant in *Aloe*. Calcium, iron, copper, magnesium, chromium, potassium, manganese, sodium, phosphorus, and zinc are significant minerals extracted from *Aloe vera* (http://wholeleaf.com, accessed 24 October 2022). *Aloe vera* gel is mostly composed of cellulose, mannose-containing polysaccharides, and pectic polysaccharides. Other phytochemicals include enzymes, lectins, anthrones, polymannans, resins, sterols, acetylated acids, terpenoids, tannins, mannan compounds, and flavonoids [49,50,51,52,53,54]. In the current review, we have detailed the effects of *Aloe vera* as alternative to antibiotics on growth performance, antimicrobial and antiparasitic activities and blood biochemical alternations in poultry (Figure 2).

## 2. Growth Effects in Poultry

Improved growth promoting effects in term of feed intake, weight gain and feed efficiency of *Aloe vera* has been documented (Table 1). According to Singh et al. [55], *Aloe vera* has the potential to be a growth promoter in broiler chicks, and its growth-stimulating effects are comparable to antibiotic growth promoters. Mmereole [56] evaluated the growth-promoting effects of *Aloe vera* and terramysin in broiler and discovered that the former treatment caused chickens to gain weight at a considerably greater rate. In broilers dosed with water-based infusions of polyherbal plants, including *Aloe vera* (20 mL/L of drinking water), Raziq et al. [57] observed better feed efficiency and weight growth. *Aloe vera* gel extract in drinking water is more effective in enhancing broiler performance than antibiotic growth promoters, according to Bernard et al. [58] and Nalge et al. [59], without having a negative impact on the birds’ general health status. Enhancing feed intake, endogenous digestive enzyme secretion, antioxidation status, and antibacterial properties are a few positive effects demonstrated by *Aloe vera* [60,61]. Improved growth effects have also been positively correlated with positive effects on intestinal histological features in broilers and other poultry species (Table 1), as reported elsewhere [62,63,64,65].

Poults administered with 30 mL/L *Aloe vera* gel, Bolu et al. [63] found that the growth metrics, such as weight increase and feed conversion efficiency, were considerably greater. According to Fallah [78], broilers fed a diet containing 1.5% garlic powder and 1.5% *Aloe vera* gel had the greatest final body weights, the highest feed consumption, and the lowest FCR. *Aloe vera* supplementation for broilers boosted body weight gain, feed efficiency, and lowered feed consumption, according to studies by Sinurat et al. [92] and Akram et al. [93]. *Aloe vera* powder used as a dietary supplement at a rate of 1.5% was more effective than the antibiotic growth promoter enramycin at enhancing broiler performance and lowering intestinal *Salmonella* and *Escherichia coli* species [72,94,95,96]. *Aloe vera*’s ability to increase the quantity of beneficial bacteria while reduce the level of detrimental bacteria can be credited with the improved growth performance. In this regard, Shokraneh and Ghalamkari [77] came to the conclusion that broilers treated with *Aloe vera* in drinking water exhibited an increase in *Lactobacillus* bacteria, which are well-known for their function in promoting nutrient digestion and absorption in the gut of birds. As a result, there are less detrimental bacteria present, which allows the birds to use the feed and nutrients more effectively. *Aloe vera*’s composition, which is rich in polysaccharides, vitamins, minerals, and organic acids [97], improve feed consumption, and consequently, the growth rate is higher.

Potassium, iron, chromium, magnesium, sodium, copper, calcium, zinc, and manganese are just a few of the minerals and vitamins that may be found naturally in *Aloe vera* [98]. These minerals are fundamental for the synthesis of the endogenous enzymes that help in nutrient digestion [99]. In addition, the plant itself has a lot of enzymes, which aid in the body’s absorption of essential nutrients [100]. Amylase is one of the primary enzymes, and the plant also has oxidase, carboxypeptidase, and alkaline phosphatase as well as isozymes of superoxide dismutase, carboxypeptidase, and glutathione peroxidase, which are proteolytic enzymes [101]. All these enzymes help in metabolism.

According to Darabighane et al. [62], the 2% *Aloe vera* gel group had increased body weight gain and feed intake that was more than the antibiotic group in a non-significant way (virginiamycin). *Aloe vera* supplementation in broilers reportedly reduced feed consumption, according to some authors [92,93]. Changes in feed taste and increased appetite can be linked to increased feed intake in *Aloe vera* gel supplemented birds [102]. Wenk (2002) added that herbs can increase endogenous secretions and hunger, which in turn can enhance performance. Studies on broilers have revealed that 600 mg of *Aloe vera* gel water extract causes broilers’ body weight to increase significantly in the third and sixth weeks when compared to the control group [70]. There might be a number of causes for the increase in the productive performance of chickens fed *Aloe vera*. First, anthraquinones and its derivatives, including isobarbaloin, aloe-emodin-9-anthrone, and anthrone-C-glycosides, are present in the phenolic components [1,64,67,97]. These compounds function as potent antibacterial agents and enhance nutrition absorption from the colon. By breaking down carbohydrates and fats, bio-catalysts like lipase and amylase found in plants can aid in digestion [64,73,97]. However, it should be noted that, while investigating the effects of herbal supplements in broiler feed on performance, various factors, including plant parts, physical characteristics, genetic variation, age, various dosages used, extraction method, harvest time, and compatibility with other ingredients, can have a different impact on performance [103,104]. In addition to plants and their extracts, researchers also focused on the polysaccharides found in herbs. Several studies on these compounds indicate that the polysaccharides in herbs and mushrooms have immunomodulatory and, in some cases, even antibacterial effects [105,106]. Such performance-enhancing benefits of herbs and polysaccharides are due to impacts on immune system activation, which leads to a decrease in bacterial and viral infections [107]. According to some experts, herbal medicines have comparable qualities to prebiotics [108,109,110]. Since the primary polysaccharide in *Aloe vera* gel is acemannan, the increased body weight gain in *Aloe vera* gel-treated groups compared to the control group may be explained by the *Aloe vera* gel’s antibacterial capabilities, which might boost gut microbiota. Additionally, the acemannan in *Aloe vera* gel helps boost the immune system and increase the body’s resilience to viruses and germs [97].

## 3. Immunomodulatory and Antimicrobial Effects

*Aloe vera* has powerful phytochemical activity, which has the ability to significantly enhance immune function by improving humoral and cellular responses [87]. In Fayoumi chicks between 28–58 days of age, Khan et al. [79] demonstrated that adding leaf powder (1% or 2% in diet) boosted haemagglutination inhibition titres. A 42-day supplementation of *Aloe vera* powder (2.5, 5, and 7.5 g/kg) increased humoral responses in broiler against common infectious diseases [111]. In response to feeding 0.1% or 0.2% whole plant powder in feed, increased antibody titers against the New Castle virus (NDV) were discovered in 42-day-old broilers [82]. Birds receiving 1% *Aloe vera* gel in their drinking water exhibited better antibody titres against sheep red blood cells (SRBCs) and NDV, as well as a stronger reaction to a phytohemagglutinin-P (PHA-P) injection, as compared to a control group, according to research by Darabighane et al. [112]. In broiler chicks (1 to 42 days of age), diets supplemented with 2% *Aloe vera* gel increased the cellular immunological response to PHA-P injections compared to a control group [84]. According to Akhtar et al. [86], consumption of an aqueous and ethanolic *Aloe vera* extract increased chicken antibody titres against SRBCs. Studies have indicated that acemannan can cause macrophages to produce inflammatory cytokines such as the tumour necrosis factor (TNF), interleukin-1 (IL-1), and IL-6, which can increase the number of T lymphocytes and encourage the proliferation of B lymphocytes [113,114]. Certain substances, such as acemannan present in *Aloe vera* gel, can trigger antibodies and cytokines and enhance the activity of natural killer cells and lymphocytes, which are responsible for this enhancement in humoral immunity [79]. According to Zhang and Tizard [113], acemannan, which raises cytokines, activates macrophages, and produces nitric oxide, is responsible for the beneficial effects of *Aloe vera* supplementation on immunity. However, the antibacterial properties of *Aloe vera* may be responsible for improving the gut flora and the ensuing improvement in immunity.

Numerous studies have shown that plant extracts have antibacterial capabilities that can improve the environment of the digestive system by lowering some diseases and enhancing gut flora populations [115]. As it contains anthraquinones, which are comparable to prebiotics in that they increase the *Lactobacillus* spp. colonies and decrease Gram-negative bacteria, *Aloe vera* has action against harmful bacteria like *Staphylococcus aureus* and *Escherichia coli* [84]. Treatment of birds with 0.75% or 1% *Aloe vera* gel aqueous extract in drinking water decreased *Coliform* spp. numbers and boosted *Lactobacilli* spp. populations [77]. *Aloe vera* includes salicylic acid, which has anti-inflammatory and antibacterial activities [99], and its leaves contain saponins and active anthraquinones [51]. Acemannan induces macrophages to produce inflammatory cytokines, which has an indirect antimicrobial effect [113]. According to Islam et al. [73], broilers fed *Aloe vera* had lower faecal *E. coli* and *Salmonella* populations than the control group, but there was no difference in the overall bacterial count. Amaechi and Iheanetu [116] also observed that feeding *Aloe vera* to broilers dramatically reduced the number of faecal bacteria. *Aloe vera*’s antibacterial qualities can enhance gut microflora. Additionally, the acemannan in *Aloe vera* boosted the immune system, increased bodily defence against germs, and altered intestine shape, which indirectly impacted growth performance [103].

## 4. Antiparasitic Effects

Coccidiosis is a frequent and deadly parasitic disease that affects poultry, especially those kept in deep-litter systems. Aloes have long been utilised for a variety of medicinal purposes, including the treatment of parasite problems. The most expensive and pervasive parasitic disease affecting the poultry business is avian coccidiosis, which has primarily been managed with the use of chemotherapy. Alternative control strategies are needed since drug-resistant bacteria have emerged [91].

According to Yim et al. [91], all treatment groups that received *Aloe vera* supplements showed considerably lower faecal oocyst shedding than the control group. Additionally, after infection, the *Aloe vera*-supplemented group had noticeably fewer intestinal lesions than the unsupplemented group. The results of Desalegn and Ahmed’s study [117] showed that different concentrations of crude aloe gel from *Aloe debrana* and *Aloe pulcherrima* have anticoccidial action as shown by their capacity to greatly reduce the sporulation of unsporulated *Eimeria* oocysts in comparison to the control. According to this study, *Aloe* gel infusions can stop oocysts from developing into the infectious stage of the *Eimeria* parasite or can at least stop them from growing (sporozoites). The antioxidant phytochemicals of the polysaccharide derivatives in *Aloe* gel may have exhibited an antisporulation effect by interfering with physiological processes required for sporulation, such as preventing oxygen access (inhibiting cells’ oxygen consumption) and inhibiting various sporulation-related enzymes [117]. Aloe gel’s anticoccidial property and saponin content effect on protozoan growth by reacting with cholesterol on the parasitic cell membrane and causing parasitic death, which is thought to be the cause of the anticoccidial action [118].

In the literature, treatment of birds with *Aloe vera* with and without coccidial infection has shown conflicting outcomes [72,76,77,91]. There are several medical applications for Aloe gel, including the treatment of digestive issues [67]. Additionally, it lowers inflammation and has favourable effects on microbiota. According to Surjushe et al. [51], *Aloe vera* contains polysaccharide (glucomannan) and growth hormones (auxins and gibberellins) that lessen the inflammation and haemorrhages in the intestines brought on by Eimeria species, improving digestion and promoting weight gain. *Aloe vera*’s beneficial effects may be related to its antioxidant activity, which may lessen the intensity of the infection [119]. Since *Aloe vera* contains active substances like beta-carotene, folic acid, choline, vitamins C and E, vitamin B12, acemannans, glucomannans, proteins, and minerals that effectively control the multiplication of oocysts in the intestines and caeca of birds, led to the conclusion that *Aloe vera* is beneficial against a variety of diseases [51]. As its gel includes anthraquinone derivatives, including isobarbaloin, aloetic acid, and emodin, *Aloe vera* may be helpful to reduce coccidia [50]. These substances work with the gastrointestinal mucosa and promote peristalsis to speed up the release of coccidiosis from faeces. According to Darabighane and Nahashon [120], feed containing *Aloe vera* gel enhanced intestinal health and controlled coccidiosis, albeit the precise dosage and degree of challenge must yet be determined. According to Akhtar et al. [86], both aqueous and ethanolic *Aloe vera* extracts have the potential to be effective immunotherapeutic agents against coccidiosis in the poultry industry.

## 5. Hematological and Serum Biochemical Effects

Blood biochemical and haematological characteristics can serve as a gauge for the bird’s internal environment. It has been suggested that *Aloe vera* improves the passage of nutrients and oxygen into cells [99]. Rehman et al. [121] observed that the timing of the administration of multiple herbal plants, including *Aloe vera*, affected the cholesterol profile of broiler chicks. They came to the conclusion that water-based infusion at a rate of 10 mL/L of drinking water every other day decreased total cholesterol, triglycerides, LDL, and VLDL, as well as total cholesterol to HDL ratios, LDL to HDL ratios, total cholesterol to VLDL. Raziq et al. [57] found that water-based infusion of polyherbal plants containing *Aloe barbadensis* increased HDL concentration and decreased blood cholesterol, triglyceride, LDL in broiler chicks. Singh et al. [55] and Yadav et al. [88] reported that a significant increase in Hb and RBCs values in chicks supplemented with *Aloe vera* juice in drinking water was seen as compared to control groups. Mmereole [56,56], Mahdavi et al. [122] and Yadav et al. [88] reported a significant increase in hematological values for mean corpuscular haemoglobin concentration (MCHC) and highest RBCs and mean corpuscular haemoglobin (MCH) values were observed in *Aloe vera* supplemented birds compared with the control group. Singh et al. [55] demonstrated that *Aloe vera* contains different active compounds (glucomannans, acemannans, carotene, and vitamin B12) which help in boosting the total leucocytes count (TLCs). In comparison to the control group that did not receive any supplements, Taraneh [123] found that broilers (42 days old) received 3% *Aloe vera* gel in their drinking water had higher blood total protein levels and lower concentrations of uric acid, cholesterol, glucose, low-density lipoprotein (LDL), and triglycerides. In comparison to a control group, broiler chicks that received a whole leaf extract in drinking water (20 g/L) daily from 1 to 42 days of age exhibited increased haemoglobin, packed cell volume, serum calcium values, and total plasma glucose levels. *Aloe vera* leaf powder supplementation at a dosage of 0.5% had no effect on the total leucocyte count, packed cell volume (PCV), haemoglobin (Hb), RBC, serum albumin, total protein, globulin, triglycerides, and glucose in the blood of Japanese quail at 35 days of age [89]. However, triglycerides, cholesterol, and glucose levels in broiler blood at 42 days of age were not affected by dietary supplementation with *Aloe vera* powder (0.1 or 0.2%) [82]. At 35 days of age, there were no appreciable differences in serum levels of the enzymes alanine aminotransferase (ALT), aspartate aminotransferase (AST), and alkaline phosphatise (ALP), markers of antioxidant status and liver injury in Japanese quail [90]. However, broiler chicken fed with gel (1.5 or 3.0%) in the drinking water at 42 days of age showed decreased blood activity of ALP, AST, and ALT when compared to a control, according to Fallah [78]. *Aloe vera* leaves alcoholic extract (2, 5 or 7 g/kg diet) supplementation in the diet of Jabalpur colour birds (32 weeks of age) lowered lipid peroxidation, enhanced antioxidant status, and offered protection to the liver and kidney, according to Sinha et al. [87]. *Aloe vera* is a natural antioxidant that may be used as a great alternative to synthetic antioxidants since it is effective at preventing cellular and membrane damage brought on by pro-oxidants. By promoting the activity of endogenous antioxidant enzymes, including catalase, glucose-6-phosphate dehydrogenase, and superoxide dismutase, it strengthens the body’s defences against oxidative stress [87].

## 6. Conclusions

The identification of the active components is crucial for the appropriate use of the medicinal plants since levels and kinds of constituents vary according to geographic area, variety, or origin in both consistency and variances. Depending on the plant component (leaves or gel), type (gel, powder, extract (methanolic, aqueous, or ethanolic), and dose employed, there are documented benefits of *Aloe vera* to supplementing poultry diets. Its usage as a feed additive in poultry can improve the bird’s physiological and productive performance. Because of its antibacterial, anticoccidial and immunomodulatory properties, *Aloe vera* can enhance intestinal health and performance. For the proper usage of *Aloe vera* in the poultry industry, studies targeted at understanding mechanism of action, effective forms, and dosage levels (in feed and drinking water) are required. *Aloe vera* has excellent potential for enhancing the growth performance in meat type birds, however we could not reference the egg production and quality in commercial laying hens. The benefits of adding *Aloe vera* to broiler feeds relies on a number of variables, including the dose, the contents of the diet, the genetics of the broilers, the type of application (powder, gel, extract (ethanolic or aqueous), and polysaccharide derived from gel), and many other factors.

## Figures and Tables

**Table 1:** Main effects of *Aloe vera* supplementation on poultry production and health.

	Dose and Application Method	Main Effect	Poultry Species	References
Feed intake	0.5% leaf powder	Increase	Japanese quails	Arif et al. [66]
	2% *Aloe vera* extract	Increase	Broiler	Darabighane et al. [67]
	2.5 g/L powder	Increase	Broilers	Ahmad et al. [64]
	15% *Aloe vera* powder	Increase	Satpuda poultry	Bhargande et al. [68]
	1, 1.5 and 2 g/kg *Aloe vera* powder	Increase	Vanaraja birds	Lotha and Vidyarthi [69]
Weight gain	0.5% leaf powder	Increase	Japanese quails	Arif et al. [66]
	600 mg aqueous *Aloe vera*	Increase	Broilers	Changkang et al. [70]
	1% *Aloe vera* powder	Increase	Broiler	Mmereole [56]
	6% *Aloe vera* powder	Increase	Naj quails	Ghasemi-Sadabadi et al. [71]
	2% *Aloe vera* leaves extract	Increase	Broiler	Darabighane et al. [67]
	1.5% water extract	Increase	Broilers	Amber et al. [1]
	0.1, 0.2 and 0.3% leaf powder	Increase	Broilers	Singh et al. [72]
	50 mg/L methanolic extract	Increase	Broilers	Nalge et al. [59]
	5 mL and 10 mL/L gel	Increase	Broilers	Islam et al. [73]
	2.5 and 5 g/L gel	Increase	Broilers	Ahmad et al. [64]
	1, 1.5 and 2 g/kg *Aloe vera* powder	Increase	Vanaraja birds	Lotha and Vidyarthi [69]
	10% aquous extract	Increase	Broilers	Zayed et al. [65]
	100, 200 and 300 mg/kg polysacherides	Increase	Broilers	Khaliq et al. [74]
	2 mL/L of water (leaf extract)	Increase	Turkey poults	Bolu et al. [63]
Feed conversion ratio	25 mL water extract	Improve	Japanese quails	Bejar [75]
	1.5% water extract	Increase	Broilers	Amber et al. [1]
	15.% *Aloe vera* powder	Increase	Satpuda poultry	Bhargande et al. [68]
	15 mL/L gel	Increase	Broilers	Islam et al. [76]
	0.5 and 7.5% extract	Increase	Broilers	Shokraneh et al. [77]
	1.5% gel in drinking water	Increase	Broilers	Fallah [78]
	1 and 2% leaves extract	Increase	Fayoumi chicks	Khan et al. [79]
	2.5 and 5 g/L	Increase	Broilers	Ahmad et al. [64]
	1 or 2% leaves extract	Increase	Fayoumi chickens	Olupona et al. [80]
Immune response and antimicrobial activity	1% in drinking water	Increase response to sheep red blood cells, NDV and phytohaemagglutinin-P injection	Broilers	Darabighane et al. [67]
	5 mL/L *Aloe vera* gel	Increase antibody titre against ND	Broilers	Islam et al. [73]
	1 or 2% leaves extract	Increased haemagglutination inhibition titres	Fayoumi chicks	Khan et al. [79]
	50, 100 or 150 mg/L	Increased humoral responses against viral infections	Broiler	Ojiezeh and Eghafona [81]
	1% *Aloe vera* gel	Improved antibody titres against sheep RBCs	Broilers	Darabighane et al. [67]
	0.1 or 0.2% *Aloe vera* powder	Improved antibody titer against NDV	Broilers	Mehala and Moorthy [82]
	0.75% or 1% Aloe vera gel	Reduced *Coliform* spp. and increased *Lactobacilli* spp.	Broilers	Shokraneh et al. [77]
	5 mL and 10 mL/L gel	Reduced in fecal *E. coli* and *Salmonella* population	Broilers	Islam et al. [73]
	100 to 300 ppm extract	Decreased aflatoxin B1 in egg yolk	Laying hens	Mohajer et al. [83]
	1.5, 2.0 and 2.5%	Increased *Lactobacillus* count and decreased *E. coli* count	Broilers	Darabighane et al. [84]
	0.1% *Aloe vera* gel	Reduced *E. coli* count	Broilers	Dai et al. [85]
	300 mg/kg ethanolic aqueous extract	Increased antibody response against RBCs	Broilers	Akhtar et al. [86]
Antioxidant capacity	1.5% water extract	Increase TAC, GPx and decrease MDA	Broilers	Amber et al. [1]
	2, 5 and 7 g/kg alcoholic extract	Increase antioxidant status and decrease lipid peroxidation	Jabalpur color birds	Sinha et al. [87]
	300 ppm extract	Decreased lipid peroxidation in egg yolk	Laying hens	Mohajer et al. [83]
Hematology and blood biochemistry	0.5% leaf powder	Increase HDL	Japanese quails	Arif et al. [66]
	1.5% water extract	Decrease triglycerides, total cholesterol, and LDL	Broilers	Amber et al. [1]
	1, 1.5 and 2 g/kg *Aloe vera* powder	No effect on cholesterol, RBC, WBC, PCV, Hb	Vanaraja birds	Lotha and Vidyarthi [69]
	10% aqueous extract	Increase Hb, RBC	Broilers	Zayed et al. [65]
	1.5% water extract	Increase Protein and albumin	Broilers	Amber et al. [1]
	1.5 or 3% aqueous solution of Alo vera	Decrease AST, ALT and ALP	Broiler	Fallah [78]
	10% aqueous solution of *Aloe vera*	Increase Hb	Broiler	Zayed et al. [65]
	0.5% *Aloe vera* powder	Increase WBCs	Broiler	Singh et al. [55]
	0.2% *Aloe vera* extract	Increase	Broiler	Yadav et al. [88]
	0.5% powder	No significant effect on AST, ALT, Hb, total protein, albumin, globulin, glucose	Broilers	Tariq et al. [89,90]
	0.1 and 0.2% powder	No effect on triglyceride, glucose and cholesterol	Broilers	Mehala and Moorthy [82]
	2.5 and 5 g/L gel	Heterophils, monocytes, eosinophils, lymphocytes	Broilers	Amber et al. [1]
	5 mL and 10 mL/L gel	No effect on WBCs, RBCs, Hb, Hct	Broilers	Islam et al. [73]
Anticoccidial effect	2.5 and 5 g/L	Reduction in cecal occysts, lesion score, and intestinal damage	Broilers	Ahmad et al. [64]
	0.1, 0.3 and 0.5% powder	Less fecal shedding compared to control	Broilers	Yim et al. [91]
	300 mg/kg ethanolic aqueous extract	Lower mean lesion score in ceca	Broilers	Akhtar et al. [86]
	2.5% gel	Smallest fecal oocyst shedding	Broilers	Darabighane and Zarei [62]
Histological features	2% *Aloe vera* gel	Higher villus height and villus height to crypt depth ratio	Broiler	Darabighane et al. [67]
	10% *Aloe vera* aqueous extract	Increased villus height	Broilers	Zayed et al. [65]
	2.5 and 5g/L *Aloe vera* gel	Restoration of intestinal villi under coccidial challenge	Broilers	Ahmad et al. [64]
	2 mL/L of water leaf extract	Normal histology of ileum, spleen, liver, and breast muscle under *E. coli* challenge	Turkey poults	Bolu et al. [63]

NDV: New Castle Disease Virus; TAC: Total Antioxidant Capacity; GPx: Glutathione peroxidase; MDA: Malanodialdehyde; HDL: High density lipoproteins; LDL: Low density lipoproteins; RBC: Red blood cells; WBC: White blood cells; PCV: packed cell volume; Hb: hemoglobin; AST: aspartate amino transferase; ALT: Alanine amino transferase; ALP: Alkaline phosphatase.

## Data Availability

Not applicable.
